# Clinical Characteristics of Japanese Patients Who Visited a COVID-19 Aftercare Clinic for Post-Acute Sequelae of COVID-19/Long COVID

**DOI:** 10.7759/cureus.18568

**Published:** 2021-10-07

**Authors:** Yuki Otsuka, Kazuki Tokumasu, Yasuhiro Nakano, Hiroyuki Honda, Yasue Sakurada, Naruhiko Sunada, Daisuke Omura, Kou Hasegawa, Hideharu Hagiya, Mikako Obika, Keigo Ueda, Hitomi Kataoka, Fumio Otsuka

**Affiliations:** 1 Department of General Medicine, Okayama University Graduate School of Medicine, Dentistry and Pharmaceutical Sciences, Okayama, JPN

**Keywords:** long covid, covid-19 aftercare clinic, general fatigue, general physician, post-acute sequelae of covid-19

## Abstract

Introduction

The long-term clinical course, prognosis, and optimal management of symptoms and conditions after the acute phase of coronavirus disease 2019 (COVID-19) remain to be elucidated. The purpose of this study was to clarify the characteristics of patients referred to a COVID-19 aftercare (CAC) clinic established at a tertiary academic hospital in Japan.

Methods

This study was a descriptive case series study. All patients who visited the CAC clinic between February 15 and September 17 in 2021 were included. Patients’ background, chief complaints, and clinical courses after the onset of COVID-19 were described.

Results

A total of 87 Japanese patients (median age, 40.0 years; interquartile range [IQR], 26.5-53.0 years; 52.9% women) were referred to the CAC clinic. The median interval between the onset of COVID-19 and the visit to the clinic was 79.0 (IQR, 52.5-112.0) days. Referral sources were hospitals (36 patients), clinics (47 patients), a local healthcare center (3 patients), and other (1 patient). The most common chief complaint was general fatigue (50.4%) followed by dysosmia (28.7%), dysgeusia (26.4%), hair loss (18.4%), headache (17.2%), dyspnea (16.1%), and dyssomnia (13.1%). Respiratory symptoms were common in the early stages of the disease but were less common as the chief complaints when visiting the clinic. On the other hand, neurological, psychiatric, and extremity symptoms were predominant one month after the onset of COVID-19.

Conclusions

Regardless of the severity in the acute phase, patients visiting our CAC clinic suffered from a variety of symptoms. General physicians skilled in using a comprehensive approach would be optimal to see patients with such complex symptoms.

## Introduction

As of September 2021, a year and a half have passed since the beginning of the novel coronavirus disease 2019 (COVID-19) pandemic. Studies have revealed that more than one-third of COVID-19 patients suffer from a range of persistent symptoms, including fatigue, headache, breathlessness, hair loss, and impaired sense of taste and smell, after the acute phase of the infection [1-2]. When these manifestations remain for more than four weeks after the onset of COVID-19, they are currently termed post-acute sequelae of COVID-19 (PASC) or long COVID [2-3]. Most cohort studies have shown that general malaise is the most common symptom, accounting for about half of the cases, followed by dyspnea and headache, which usually last up to three or four months after the onset of the disease [2]. Although information on these unique conditions has been accumulating, their long-term clinical courses, treatment options, and prognosis remain to be elucidated.

Due to the increasing number of cases of PASC/long COVID in Japan, we established a clinical center for such patients in our university hospital, named the COVID-19 aftercare (CAC) clinic, in February 2021. The CAC clinic has been widely publicized throughout Japan, and the clinic accepts patient referrals from general practitioners, community hospitals, and a regional healthcare center. The purpose of this study was to clarify the clinical characteristics of patients referred to the CAC clinic.

## Materials and methods

This study was a descriptive study conducted in a single facility. The CAC clinic was opened in February 2021 in the department of general medicine, Okayama University Hospital, a tertiary hospital with 865 beds located in the western area of Japan. Physicians working in the clinic have diverse specialties, including primary care, psychiatry, endocrine diseases, infectious diseases, and Japanese traditional (Kampo) medicine. The CAC clinic accepts patients referred for persistent symptoms even one month (28 days) after the onset of COVID-19, and the clinic collaborates with other departments such as the psychiatry, dermatology, and otolaryngology departments.

All patients who visited the CAC clinic during a period of seven months from February 15 to September 17 in 2021 were included in this study. We reviewed the medical records of the patients and obtained information on each patients’ age, gender, the clinical course of acute COVID-19, chief complaints and symptoms, treatment history, and referral source hospital. Information on the medical treatment given and the clinical course after the visitation was not included.

The present study was approved by the Ethical Committee of Okayama University (No. 2105-030) and adhered to the Declaration of Helsinki. An announcement on this study was provided on the website [4] and inside the clinic, and a meeting point was provided for patients who wished to opt out.

## Results

A total of 87 Japanese patients, including 41 men (47.1%) and 46 women (52.9%), with a median age of 40.0 years (interquartile range [IQR], 26.5-53.0 years) were referred to the CAC clinic during the study period. The median body mass index was 22.5 (IQR, 20.5-26.6). They had medical histories of lung diseases, including asthma (12 patients, 13.8%), cardiovascular diseases (6 patients, 6.9%), hypertension (14 patients, 16.1%), diabetes (4 patients, 4.6%), or neuropsychiatric diseases (8 patients, 9.2%).

Only 34 patients (33.0%) had been hospitalized during the acute phase, and 13 (12.6%) of those patients required oxygen administration. Only three patients (2.9%) required admission to the intensive care unit and received mechanical ventilation. The median duration of hospitalization was 12 days (IQR, 9.0-18.3 days) for the remaining 32 patients, excluding two patients whose exact discharge date was not obtained. The median interval between the onset of COVID-19 and visit to the CAC clinic was 79.0 days (IQR, 52.5-112.0 days). Referral sources included hospitals (36 patients, 41.2%), clinics (47 patients, 54.0%), a local health center (3 patients, 3.4%), and the other; nursing school of the university hospital (1 patient, 1.1%) (Table 1).

**Table 1:** Patients' background

Age	
Median (IQR)	40.0 ( 26.5-53.0 )
Age group	
-19	9 (10.3%)
20 - 29	20 (23.0%)
30 - 39	14 (16.1%)
40 - 49	15 (17.2%)
50 - 59	20 (23.0%)
60 - 69	5 (5.7%)
70 -	4 (4.6%)
Sex	
Male	41 (47.1%)
Female	46 (52.9%)
Past medical history	
Lung diseases	12 (13.8%)
Cardiovascular diseases	6 (6.9%)
Hypertension	14 (16.1%)
Diabetes	4 (4.6%)
Neuropsychiatric diseases	8 (9.2%)
Referral source	
From hospitals	36 (41.2%)
From clinics	47 (54.0%)
From a local health center	3 (3.4%)
Acute phase care	
Hospitalized	34 (33.0%)
Oxygen administration	13 (12.6%)
Intensive care	3 (2.9%)
At accommodation facilities	18 (17.5%)
At home	35 (34.0%)
Visitation days after the onset of COVID-19	
28 - 56	26 (29.9%)
56 - 84	20 (23.0%)
84 - 112	19 (21.8%)
112 - 140	9 (10.3%)
140 - 168	2 (2.3%)
168 -	11 (12.6%)

**Figure 1:**
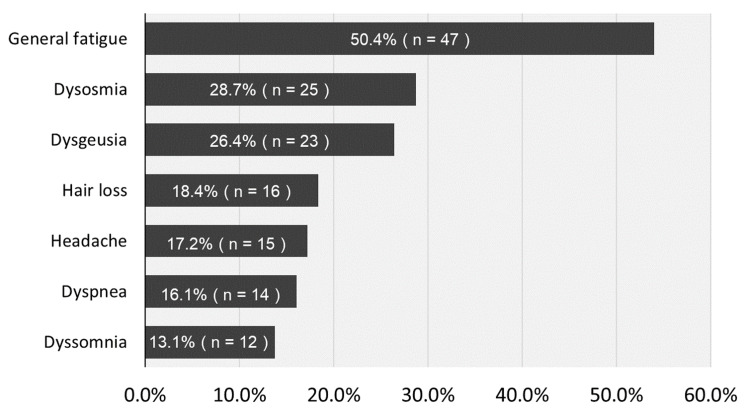
Percentage of common chief complaints for visiting the CAC clinic The most common complaint was general fatigue (47 patients, 50.4%) followed by dysosmia (25, 28.7%), dysgeusia (23, 26.4%), hair loss (16, 18.4%), headache (15, 17.2%), dyspnea (14, 16.1%), and dyssomnia (12, 13.1%). CAC: COVID-19 aftercare

Figure 1 shows the percentage of common chief complaints for visiting the CAC clinic. The most common complaint was general fatigue (47 patients, 50.4%) followed by dysosmia (25, 28.7%), dysgeusia (23, 26.4%), hair loss (16, 18.4%), headache (15, 17.2%), dyspnea (14, 16.1%), and dyssomnia (12, 13.1%).

The clinical courses of each symptom by time period before visiting the clinic are shown in Table 2. More than half of the patients experienced weight loss, fever, general fatigue, cough, sore throat, dyspnea, loss of appetite, dysgeusia, dysosmia, headache, dyssomnia, and anxiety sometime in the whole course of the disease. Not all of these common symptoms during the clinical course coincided with the main reasons for visiting the CAC clinic, which showed in Figure 1. General fatigue, dyspnea, dysgeusia, dysosmia, headache, dyssomnia, and anxiety were common symptoms throughout the time period from the acute to the chronic phase. Respiratory and gastrointestinal symptoms were more common in the early stages of the disease while hair loss and irritability tended to become an apparent chronic phase of COVID-19.

**Table 2:** Clinical courses of each symptom by time period from the onset of COVID-19 More than half of the patients experienced weight loss, fever, general fatigue, cough, sore throat, dyspnea, loss of appetite, dysgeusia, dysosmia, headache, dyssomnia, and anxiety sometime in the whole course of the disease. General fatigue, dyspnea, dysgeusia, dysosmia, headache, dyssomnia, and anxiety were common symptoms throughout the time period from the acute to the chronic phase. Respiratory and gastrointestinal symptoms were more common in the early stages of the disease while hair loss and irritability tended to become apparent in the chronic phase of COVID-19.

	Symptom presence in whole period		Symptoms by period after COVID-19 onset				
			0 - 14 days	14 - 28 days	28 - 56 days	56 - 84 days	84 - 112 days
	( n = 87 )		( n = 87 )	( n = 87)	( n = 61 )	( n = 41 )	( n = 22 )
General symptoms							
Weight loss	46	( 52.9% )	34.5%	19.5%	23.0%	12.2%	9.1%
Fever	48	( 55.2% )	46.0%	21.8%	16.4%	19.5%	9.1%
General fatigue	77	( 88.5% )	64.4%	55.2%	60.7%	65.9%	59.1%
Respiratory symptoms							
Nasal discharge	35	( 40.2% )	27.6%	11.5%	8.2%	4.9%	0.0%
Cough	53	( 60.9% )	50.6%	23.0%	18.0%	4.9%	4.5%
Sputum	42	( 48.3% )	34.5%	12.6%	11.5%	4.9%	4.5%
Sore throat	46	( 52.9% )	41.4%	14.9%	13.1%	9.8%	9.1%
dyspnea	53	( 60.9% )	39.1%	35.6%	27.9%	31.7%	31.8%
Thoracic symptoms							
Chest discomfort	37	( 42.5% )	23.0%	21.8%	26.2%	22.0%	22.7%
Palpitations	43	( 49.4% )	24.1%	24.1%	26.2%	34.1%	27.3%
Gastrointestinal symptoms							
Nausea	27	( 31.0% )	18.4%	9.2%	19.7%	12.2%	13.6%
Loss of appetite	50	( 57.5% )	40.2%	23.0%	14.8%	17.1%	18.2%
Constipation	27	( 31.0% )	24.1%	16.1%	9.8%	19.5%	18.2%
Diarrhea	33	( 37.9% )	32.2%	13.8%	13.1%	17.1%	9.1%
Cutaneous symptoms							
Hair loss	30	( 34.5% )	14.9%	12.6%	16.4%	26.8%	31.8%
Rash	17	( 19.5% )	14.9%	6.9%	8.2%	7.3%	9.1%
Neurological symptoms							
Dysgeusia	50	( 57.5% )	42.5%	27.6%	27.9%	24.4%	36.4%
Dysosmia	51	( 58.6% )	46.0%	32.2%	36.1%	36.6%	50.0%
Headache	50	( 57.5% )	35.6%	29.9%	36.1%	41.5%	31.8%
Dizziness	33	( 37.9% )	19.5%	12.6%	21.3%	24.4%	22.7%
Psychiatric symptoms							
Dyssomnia	53	( 60.9% )	32.2%	27.6%	37.7%	36.6%	36.4%
Irritability	36	( 41.4% )	18.4%	16.1%	29.5%	24.4%	40.9%
Anxiety	58	( 66.7% )	42.5%	33.3%	49.2%	46.3%	45.5%
Extremity symptoms							
Numbness	25	( 28.7% )	13.8%	9.2%	19.7%	22.0%	27.3%
Weakness	31	( 35.6% )	18.4%	14.9%	19.7%	24.4%	27.3%
Muscle pain	33	( 37.9% )	23.0%	10.3%	16.4%	19.5%	13.6%
Joint pain	38	( 43.7% )	28.7%	13.8%	13.1%	14.6%	9.1%

## Discussion

We determined the clinical characteristics of Japanese patients referred to the specialized outpatient clinic for PASC/long COVID. Chronic manifestations of COVID-19 have been reported in various countries in Europe [5-8], the United States [9-10], China [11], and Japan [12], and several review articles and a meta-analysis of PASC/long COVID have been published [1-2,13], and it has been shown that one-third to half of the patients with COVID-19 show residual symptoms for a relatively long duration, even up to several months. This is the first English report which described the patient’s characteristics of PASC/long COVID specialized clinic in Japan. Additionally, in contrast to most previous studies that focused on the follow-up of patients with acute COVID-19, our study is unique in that we focused on patients who were referred to a specialized clinic managing patients suffering from PASC/long COVID.

Our study revealed that general fatigue was the most common main reason for visiting the CAC clinic, which is consistent with previous studies [11,14-15]. It has been reported that general fatigue occurs in the chronic phase regardless of the severity of disease in the acute phase [11], and that seems to be consistent with our patient cohort. Management of fatigue is often challenging for both physicians and patients because fatigue is difficult to quantify and diagnose objectively, possibly complicating the physician-patient relationship [16]. In addition, fatigue is caused by a wide variety of systemic diseases other than COVID-19 [17], making a differential diagnosis difficult. A general medical approach is therefore important.

On the other hand, our study showed that respiratory symptoms were not one of the main reasons for visiting the CAC clinic despite the high frequency of such symptoms during the acute to sub-acute phases of the disease. This may be because respiratory symptoms are less likely than other manifestations to persist for more than one month. Alternatively, it might be because only a few patients with severe pneumonia were included in our study. Another possibility is the fact that patients with COVID-19 have been treated by respiratory physicians in many Japanese hospitals and their residual respiratory symptoms have therefore been managed well [18].

Our study also revealed that dysosmia, dysgeusia, and hair loss were common symptoms in patients who visited the CAC clinic. Previous studies showed that dysosmia and dysgeusia occurred in around 10% of patients after COVID-19 [11,19], however, it could be much more patients. The underlying causes of these conditions have yet to be clarified, and treatment strategies have not yet been established [20].

Most of the patients who were referred to the CAC clinic had not been hospitalized in the acute phase. This is contradictory to the results of a study showing that patients with severe pneumonia are more likely to have chronic symptoms [6]. We assume that patients with severe COVID-19 were hospitalized and could be followed up after discharge. This is supported by the fact that there were more referrals to the CAC clinic were more from outpatient clinics than from large hospitals. A recent study has shown that even young, home-isolated patients of mild COVID-19 also suffer from sequelae [8]. A patient referral system from a local healthcare center to the CAC clinic was helpful because many of the patients were young patients who had not been admitted to a hospital and did not have a primary care physician.

Several limitations of our study should also be mentioned. First, this study was a retrospective observational study conducted in a single facility without a comparison group. Further well-designed research is warranted to investigate the whole picture of the condition. Second, we did not evaluate the effectiveness of acute-phase treatment on the development and severity of PASC/long-COVID. Third, we just counted the number of chief complaints, for which we did not weigh the severity of each symptom.

Despite these limitations, we believe that the results of the present study are worthy of being shared among physicians amid the ongoing worldwide COVID-19 pandemic. It will help patients suffering from various symptoms and outpatient physicians struggling to answer their questions. We believe that the CAC clinic will become a hub for the treatment, education, and research of PACS/long COVID.

## Conclusions

We presented the clinical characteristics of patients who actually visited a COVID-19 aftercare clinic established at a university hospital in Japan. Regardless of the severity of disease in the acute phase, patients visiting our clinic suffered from a variety of PASC/long-COVID symptoms, including general fatigue, dysosmia, dysgeusia, anxiety, and hair loss. We hope to emphasize that the management of patients suffering from PASC/long COVID requires a comprehensive approach without being overly specialized for specific organs. Simultaneously, it is also necessary to consult appropriate specialists for the assessment of each specific symptom. Such an approach can be achieved by management from a combination of general physicians and specialists as in our CAC clinic. We need to continue to fight against emerging threats by utilizing optimal resources.
